# Large-scale mitogenomic analysis of the phylogeography of the Late Pleistocene cave bear

**DOI:** 10.1038/s41598-019-47073-z

**Published:** 2019-08-15

**Authors:** Joscha Gretzinger, Martyna Molak, Ella Reiter, Saskia Pfrengle, Christian Urban, Judith Neukamm, Michel Blant, Nicholas J. Conard, Christophe Cupillard, Vesna Dimitrijević, Dorothée G. Drucker, Emilia Hofman-Kamińska, Rafał Kowalczyk, Maciej T. Krajcarz, Magdalena Krajcarz, Susanne C. Münzel, Marco Peresani, Matteo Romandini, Isaac Rufí, Joaquim Soler, Gabriele Terlato, Johannes Krause, Hervé Bocherens, Verena J. Schuenemann

**Affiliations:** 1 Institute for Archaeological Sciences, University of Tübingen, Tübingen, Germany; 2 Max Planck Institute for the Science of Human History, Jena, Germany; 3 Museum and Institute of Zoology, Polish Academy of Sciences, Warsaw, Poland; 4 Institute of Evolutionary Medicine, University of Zurich, Zurich, Switzerland; 5 Swiss institute for speleology and karst studies (SISKA), La Chaux-de-Fonds, Switzerland; 6 Ancient prehistory and quaternary Ecology, University of Tübingen, Tübingen, Germany; 7 Senckenberg Centre for Human Evolution and Palaeoenvironment (S-HEP), University of Tübingen, Tübingen, Germany; 8 Service Régional de l’Archéologie de Bourgogne-Franche-Comté and Laboratoire Chronoenvironemment, CNRS, UMR, 6249 Besançon, France; 9 Laboratory for Bioarchaeology, Department of Archaeology, University of Belgrade, Belgrade, Serbia; 10 Mammal Research Institute, Polish Academy of Sciences, Białowieża, Poland; 11 Institute of Geological Sciences, Polish Academy of Sciences, Warsaw, Poland; 12 Institute of Archaeology, Nicolaus Copernicus University in Toruń, Toruń, Poland; 13 Department of humanities, Section of Prehistoric and Anthropological Sciences, University of Ferrara, Ferrara, Italy; 14 Department of Cultural Heritage, University of Bologna, Ravenna, Italy; 15 Institute of historical research, University of Girona, Girona, Spain; 16 Department of Geosciences, University of Tübingen, Tübingen, Germany

**Keywords:** Evolutionary biology, Evolutionary genetics

## Abstract

The cave bear (*Ursus spelaeus*) is one of the Late Pleistocene megafauna species that faced extinction at the end of the last ice age. Although it is represented by one of the largest fossil records in Europe and has been subject to several interdisciplinary studies including palaeogenetic research, its fate remains highly controversial. Here, we used a combination of hybridisation capture and next generation sequencing to reconstruct 59 new complete cave bear mitochondrial genomes (mtDNA) from 14 sites in Western, Central and Eastern Europe. In a Bayesian phylogenetic analysis, we compared them to 64 published cave bear mtDNA sequences to reconstruct the population dynamics and phylogeography during the Late Pleistocene. We found five major mitochondrial DNA lineages resulting in a noticeably more complex biogeography of the European lineages during the last 50,000 years than previously assumed. Furthermore, our calculated effective female population sizes suggest a drastic cave bear population decline starting around 40,000 years ago at the onset of the Aurignacian, coinciding with the spread of anatomically modern humans in Europe. Thus, our study supports a potential significant human role in the general extinction and local extirpation of the European cave bear and illuminates the fate of this megafauna species.

## Introduction

Today in the Holocene epoch, the northern hemisphere is zoologically impoverished in large terrestrial species^1,2^. Astonishingly, this is a relatively recent phenomenon. During the Late Pleistocene, until around 50,000 years ago, the continents were still populated with spectacular fauna consisting of some of the largest mammals that ever roamed the earth^2^. More than 150 genera of megafauna such as mammoths, woolly rhinoceros, and sabre-toothed cats inhabited the steppes of Eurasia and North America^1,2^. However, by 11,000 years ago, these ecosystems had lost between around 36% and 72% of their large-bodied (>45 kg) mammalian genera, respectively^3^, and at least 97 genera in total^1^. This extinction wave affecting the largest members of the herbivorous guild had cascading consequences on terrestrial ecosystems with consequences still to be seen in modern ecosystems^4–6^. Understandably, the potential causes of these incisive extinctions have remained subject to highly controversial debates. The discussed explanations include an anthropogenic contribution, climate and environmental changes or a combination of both^2,3^. However, with a growing body of data, the patterns and processes of these extinctions appear more complex. According to Lorenzen and colleagues^3^, for example, while the proportion of dwindling megafauna species was greatest on continents that underwent the most dramatic climatic and environmental changes, the extinction events in North America and Australia rather coincided with the arrival of anatomically modern humans^1,3^. The circumstances are apparently in contradiction with cross-taxa response to global climatic or anthropogenic factors, indicating a species-specific response to one or both factors. Nevertheless, recent publications promote rapid climatic shifts and oscillations, especially the Dansgaard-Oeschger warming events, as the main cause of megafauna extinctions, suggesting only a synergistic role of humans in these processes^7,8^; although this hypothesis does not receive unanimous approbation^9^. Furthermore, it was previously argued by Lorenzen and colleagues^3^ that the population development of different taxa is contingent on the geographic as well as temporal scale and the methodological approaches applied^3^. For instance, while the woolly mammoth and cave lion experienced sudden losses of genetic diversity and subsequent population stability long before their final extinction^3,10^, it was shown that genetic diversity in bison and musk ox declined gradually through the course of the Late Pleistocene^3^. The latter pattern of *withering away* was also assumed for the Pleistocene cave bear *Ursus spelaeus* sensu lato^11^. As this Late Quaternary mammal is represented by a largest fossil record in Europe^12^, the cave bear is a useful model to study the causes of the extinction of a species, especially in the context of population dynamics, climate instability and changing human impact. Descending from the Middle Pleistocene *Ursus deningeri*^13^, as indicated by morphological and molecular studies^14^, the Late Pleistocene cave bear established a vast distribution extending eastwards from Northwest Spain across Central Europe and the Urals to Arctic North-Eastern Siberia and the Altai Mountains^15,16^. Due to their high intra-specific morphological variability observed across the Eurasian cave sites, several taxonomic groups have been previously proposed mostly on the basis of morphological and metrical studies of the teeth, metapodials and the cranium^17–19^. These primarily differentiate Eurasian large-bodied cave bears from small-bodied cave bears endemic to high-altitude areas of the Alps, the Caucasus and the Altai mountains^17–19^. If these suggested morphological groupings indeed represent valid and distinct phylogenetical groups on species or subspecies level remains controversial^20^, especially since recent analyses indicate a more complex evolutionary relationship^21^. However, despite its substantial diversity and distribution, the cave bear became extinct at the beginning of the Last Glacial Maximum (LGM)^12,22–24^. The timing of its final extinction as well as the cause of the extinction, with climate change in the context of its herbivorous diet^25–28^ or human hunting impact^29^ commonly regarded as potential factors^12^, remain the subject of controversial debates. While comprehensive radiocarbon dating indicates that the extinction took place at the onset of the LGM around 28–26 ka years before present^12,22–24^, a small number of fossils younger than 26,000 calibrated years BP^23,24^ documents the survival of fragmented populations during the maximum extent of the ice sheets^30,31^. In fact, Stiller and colleagues^11^ demonstrated based on population size reconstruction that 25,000 years of genetic decline preceded not only the cave bear extinction, but also the onset of the LGM. Since this circumstance eliminates a correlation between cave bear population decline and substantial climate change, human impact, either due to direct hunting or resource competition^32–38^ emerges as the major extinction cause, albeit, archaeological evidence remains sparse for now^24,36–38^. In this context, molecular analysis of ancient DNA (aDNA) from cave bear fossils has provided substantial insights into cave bear evolution and extinction, since it allows us to identify even subtle demographic developments invisible in the palaeontological record^39^. However, previous ancient DNA studies were based on relatively small sample sizes^40^ or focused on geographically limited areas^41^. The majority of these studies was restricted to the mitochondrial D-loop sequence^42^, a 285 base pair short fragment comprising only ~1.7% of the whole bear mitogenome. As demonstrated by previous studies^43,44^, inferred genealogical reconstructions based on the D-loop region tend to contradict inferences based on the entire mitogenome. Thus, the current knowledge regarding cave bear population dynamics and phylogeography during the Late Pleistocene is substantially constrained. To overcome these limitations, here we analysed 59 new complete mitochondrial DNA sequences, representing populations from a Europe-wide time transect. Moreover, we present the first mitochondrial genome of a specimen morphologically classified as *Ursus spelaeus ladinicus* as well as the youngest cave bear mtDNA sequences thus far, which dates to 19,656 ^14^C years before present (23,907–23,461 cal. yr. BP). Our data can help to illuminate the fate of the European cave bear before its final extinction.

## Results

### Sample collection and processing

**Figure 1:**
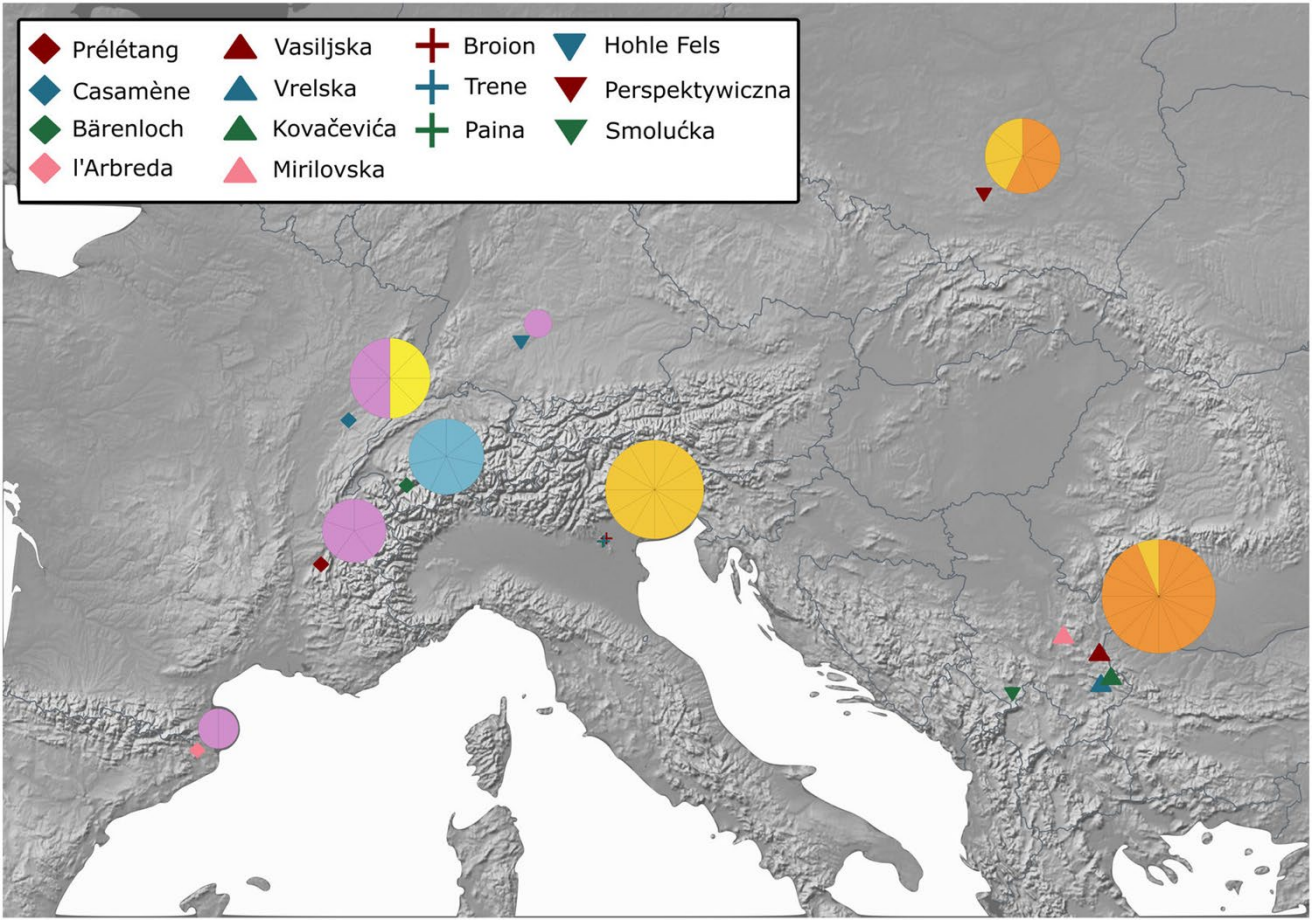
Geographical distribution of analysed samples. Circle size and number of wedges correspond to sample size, respective colours correspond to samples of a specific haplogroup: violet, *Ursus spelaeus spelaeus*; blue, *Ursus spelaeus eremus*; yellow, *Ursus ingressus* West European; light orange, *Ursus ingressus* Central European; dark orange, *Ursus ingressus* South-Eastern European.

For ancient mtDNA extraction, 81 bone specimens morphologically identified as cave bears were selected from Bärenloch (Switzerland), Perspektywiczna cave (Poland), Casamène and Prélétang (France), l’Arbreda (Spain), Hohle Fels (Germany), Broion, Paina and Trene (Italy) as well as Vrelska, Kovačevića, Vasiljska, Smolućka, and Mirilovska cave (Serbia), covering temporally spaced sites from the Iberian Peninsula to the Balkans in a time range from >49 to 23 cal. ka before present (Fig. 1, Table 1). A short description of each site can be found in Supplementary Section 1. We then used double-stranded Illumina sequencing libraries in combination with in-solution bait-capture and high-throughput sequencing to generate mitochondrial sequences for 59 of the 81 specimens. For 19 samples, it was not possible to recover sufficient amounts of aDNA to reliably infer the taxonomic position, while three individuals were subsequently identified as *Ursus arctos* during the phylogenetic analysis. The obtained mitochondrial genomes feature a coverage between 4.5 and 752.46-fold with 72%–100% of the mitochondrial genome covered (Table 1). All mitochondrial genomes exhibited C to T damage patterns indicative of authentic aDNA. The number of samples for each site producing mitochondrial genomes is as follows: Bärenloch (n = 7), Perspektywiczna Cave (n = 7); Casamène (n = 8), Prélétang (n = 5), l’Arbreda (n = 2), Hohle Fels (n = 1), Broion (n = 3), Paina (n = 5), Trene (n = 4), Vrelska cave (n = 7), Kovačevića cave (n = 7), Vasiljska cave (n = 1), Smolućka cave (n = 1), and Mirilovska cave (n = 1). Subsequently, we compared these sequences with 64 previously published complete mitochondrial genomes, resulting in an alignment of in total 123 specimens. On this final alignment, we performed Bayesian phylogenetic analysis as well as calculation of the effective female population size through time.

**Table 1:** Details of cave bear samples reported in this study. Details of Late Pleistocene European cave bear specimens successfully generating more than threefold coverage and more than 70% complete mitochondrial genomes after in-solution enrichment. Radiocar bon (^14^C) dates were calibrated to the Intcal 13 curve^74^ using the *rcarbon* package^75^. Samples without radiocarbon date are denoted by NA (not available). Radiocarbon dates obtained for this study are marked with an asterisk. Additional information about collagen quality can be found in Table S2.

ID	Site	Mean Cov.	3X Cov. in %	Classification	^14^C	Cal. ^14^C (1-sigma)
TU1	Bärenloch, CH	142.4	98.98	eremus	28,415 ± 605^76,77^	33,033–31,602
TU2	Bärenloch, CH	275.6	99.3	eremus	NA	
TU3	Bärenloch, CH	245.7	99.27	eremus	>40,000^76,77^	
TU5	Bärenloch, CH	19.7	92.21	eremus	26,745 ± 490^76,77^	31,239–30,477
TU6	Bärenloch, CH	32.4	95.59	eremus	NA	
TU7	Bärenloch, CH	130.2	98.61	eremus	NA	
TU8	Bärenloch, CH	260.3	99.08	eremus	NA	
TU76	Casamène, FR	19.2	97.59	spelaeus	NA	
TU77	Casamène, FR	79.8	100	ingressus W	47,406 ± 1309*	…−48,913
TU78	Casamène, FR	10.7	89.23	spelaeus	NA	
JK3204	Casamène, FR	100.2	99.33	spelaeus	39,456 ± 494*	43,573–42,772
JK3206	Casamène, FR	38.1	99.35	spelaeus	43,890 ± 999*	48,188–46,071
JK3212	Casamène, FR	51.2	99.82	ingressus W	38,153 ± 430*	42,608–42,039
JK3215	Casamène, FR	49.5	99.26	ingressus W	30,518 ± 170*	34,662–34,298
JK3216	Casamène, FR	80.7	99.86	ingressus W	41,366 ± 518*	45,289–44,370
JK1726	Hohle Fels, GER	16.9	97.19	spelaeus	NA	
TU151	Vrelska Cave, SRB	97.9	99.36	ingressus SE	40,470 ± 567*	44,532–43,488
TU152	Kovačevića Cave, SRB	75.4	97.8	ingressus SE	48,116 ± 1,432*	…−49,044
TU153	Vrelska Cave, SRB	16.9	90.49	ingressus C	40,595 ± 574*	44,656–43,588
TU154	Vrelska Cave, SRB	22.4	92.6	ingressus SE	45,918 ± 1,093*	…−48,390
TU155	Kovačevića Cave, SRB	125.2	98.44	ingressus SE	45,673 ± 1,067*	…−48,240
TU156	Vrelska Cave, SRB	16.2	94.37	ingressus SE	42,687 ± 740*	46,606–45,240
TU157	Vrelska Cave, SRB	363.5	99.68	ingressus SE	38,330 ± 440*	42,720–42,140
TU158	Kovačevića Cave, SRB	5.1	72.19	ingressus SE	NA	
TU163	Vrelska Cave, SRB	44.7	97.76	ingressus SE	44,748 ± 947*	49,101–47,018
TU166	Kovačevića Cave, SRB	80.2	97.65	ingressus SE	46,429 ± 1,167*	…−48,623
TU167	Vrelska Cave, SRB	9.1	87.84	ingressus SE	43,641 ± 826*	47,729–45,978
TU168	Kovačevića Cave, SRB	79.1	98.58	ingressus SE	46,376 ± 1,167*	…−48,594
TU169	Kovačevića Cave, SRB	7.3	83.21	ingressus SE	40,848 ± 590*	44,914–43,814
TU170	Kovačevića Cave, SRB	278.9	98.85	ingressus SE	45,449 ± 1,035*	49,837–47,947
TU172	Vasiljska, SRB	10.2	89.19	ingressus SE	43,027 ± 445*	46,638–45,740
TU173	Smolućka, SRB	6.7	72.59	ingressus SE	30,649 ± 113*	34,747–34,465
TU174	Mirilovska, SRB	32.7	98.68	ingressus SE	28,807 ± 149*	33,252–32,755
TU511	l’Arbreda, ES	5	73.18	spelaeus	NA	
TU512	l’Arbreda, ES	15.7	96	spelaeus	NA	
TU779	Prélétang, FR	240.7	99.58	spelaeus	NA	
TU781	Prélétang, FR	59	99.33	spelaeus	42,400 ± 409^*,^^77^	46,031–45,284
TU782	Prélétang, FR	133.7	99.45	spelaeus	40,423 ± 330^*,^^77^	44,340–43,635
TU783	Prélétang, FR	184.8	99.46	spelaeus	38,742 ± 277^*,^^77^	42,888–42,481
TU784	Prélétang, FR	324.5	99.59	spelaeus	49,788 ± 1,006^*,^^77^^,^	>45,000
TU841	Paina, IT	4.5	73.2	ingressus C	20,015 ± 46^24^	24,275–23,880
TU842	Paina, IT	17	97.34	ingressus C	NA	
TU843	Paina, IT	25.8	98.77	ingressus C	19,914 ± 45^24^	24.167–23.764
TU844	Paina, IT	6.5	87.18	ingressus C	19,975 ± 46^24^	24,234–23,839
TU847	Paina, IT	17.7	98.22	ingressus C	NA	
TU848	Broion, IT	121.5	99.67	ingressus C	29,001 ± 123^24^	33,597–32,844
TU851	Broion, IT	9.2	93.68	ingressus C	NA	
TU852	Broion, IT	6.2	84.06	ingressus C	25,978 ± 70^24^	30.630–29,855
TU853	Trene, IT	4.9	75.44	ingressus C	25,290 ± 66^24^	29,599–29,079
TU854	Trene, IT	27	98.64	ingressus C	24,755 ± 63^24^	28,977–28,566
TU855	Trene, IT	27.7	98.81	ingressus C	19,656 ± 44^24^	23,907–23,461
TU857	Trene, IT	73	99.14	ingressus C	NA	
TU860	Perspektywiczna Cave, PL	39.2	99.44	ingressus C	41,446 ± 638^*,^^28^	45,448–44,335
TU861	Perspektywiczna Cave, PL	752.5	99.74	ingressus SE	40,200 ± 1,200^28^	44,775–42,905
TU863	Perspektywiczna Cave, PL	319.5	99.73	ingressus SE	41,600 ± 1,400^28^	46,176–43,715
TU865	Perspektywiczna Cave, PL	219.2	99.72	ingressus C	47,538 ± 1,337^*,^^28^	…−48,934
TU866	Perspektywiczna Cave, PL	114.4	99.64	ingressus SE	NA	
TU867	Perspektywiczna Cave, PL	551.7	99.74	ingressus SE	NA	
TU868	Perspektywiczna Cave, PL	101.4	99.64	ingressus C	NA	

### Phylogenetic analysis

**Figure 2:**
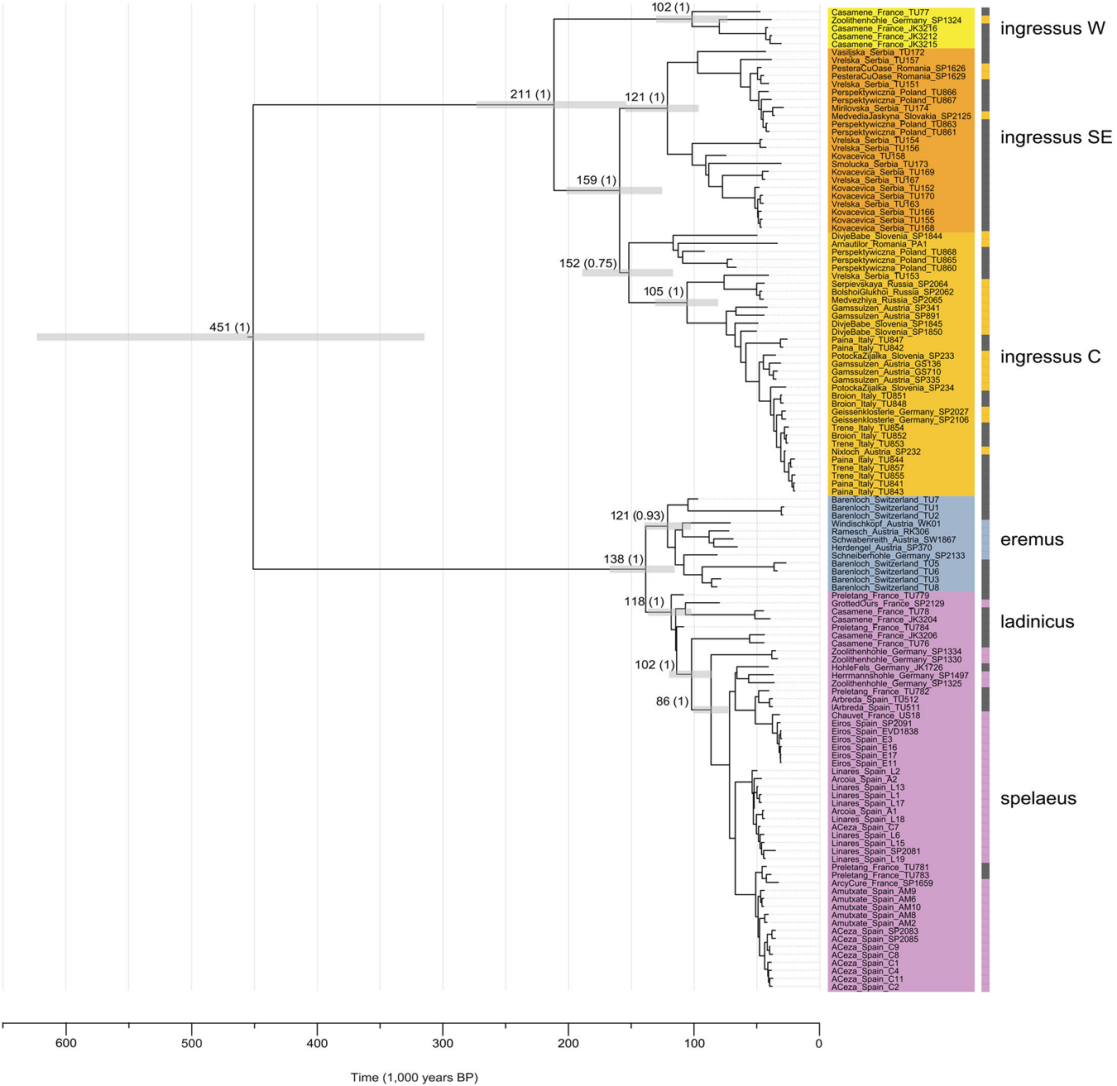
Maximum Clade Credibility (MCC) phylogeny of 123 mitochondrial genomes. MCC phylogeny resulting from a BEAST analysis of 64 previously published and 59 mitochondrial genomes reported here drawn to a timescale. The relaxed molecular clock was calibrated using the tip-dating method. Nodes leading to major clades are labelled with the inferred tMRCA (95% highest posterior density of node ages is shown by grey bars). Posterior probabilities from 200,000,000 steps are provided in parentheses along branches leading to major clades. For visibility reasons, only support values related to the relationship of the major clades are shown. Haplogroup clades^19^ are indicated by the colour coding matching Fig. 1. Haplogroup identifications^19^ based on previous mtDNA^40,41^ analyses are provided as rectangular bars to the right of sample names. Trees were visualised using Figtree 1.4.3 (tree.bio.ed.ac.uk/software/figtree/).

Our Bayesian phylogenetic analyses detected five major European lineages, sharing a most recent common ancestor (MRCA) ~451 ka BP (~314–623 ka BP 95% CI) (Fig. 2). These lineages are consistent with the major mtDNA control region haplogroups previously taxonomically designated as *Ursus ingressus* and *Ursus spelaeus* (including *U*. *s*. *eremus*, *U*. *s*. *ladinicus* and *U*. *s*. *spelaeus*). *U*. *s*. *eremus* appears to be distinct from both *U*. *s*. *spelaeus* and *U*. *s*. *ladinicus*. Samples from Prélétang, Casamène, and Grotte d’Ours assigned to the *U*. *s*. *ladinicus* control region haplogroup (Figure S1, Figure S2) form a paraphyly excluding *U*. *s*. *spelaeus*. Furthermore, we detected a noticeable subdivision of the *U*. *ingressus* clade that does not correspond to any previous classification based on morphological features. For the purpose of describing these novel groups, since there is no association with certain morphological or genetical designations, we divided the three lineages by their approximate distribution into a Western, Central and South-Eastern European group. These three groups shared a most recent common ancestor (MRCA) ~211 ka BP (~154–273 ka BP 95% CI), preceding the initial divergence of the *U*. *spelaeus* complex by roughly 73 ka (138 ka BP; 115–167 ka BP 95% CI). The most basal of these groups contains only five samples from two Western European sites, namely Casamène in France and Zoolithen cave in Germany, while the Central European and South-Eastern European group are well represented, comprising 33 samples from thirteen sites assigned to the Central European group and 23 samples from eight sites assigned to the South-Eastern European group. However, the position of the most divergent clade within the Central European Group, comprising five samples (TU860, TU865, TU868, PA1 and SP1844), is not clearly dissolved. Alternatively, it may be located ancestral to both the Central as well as the South-Eastern European group.

### Population size analysis

**Figure 3:**
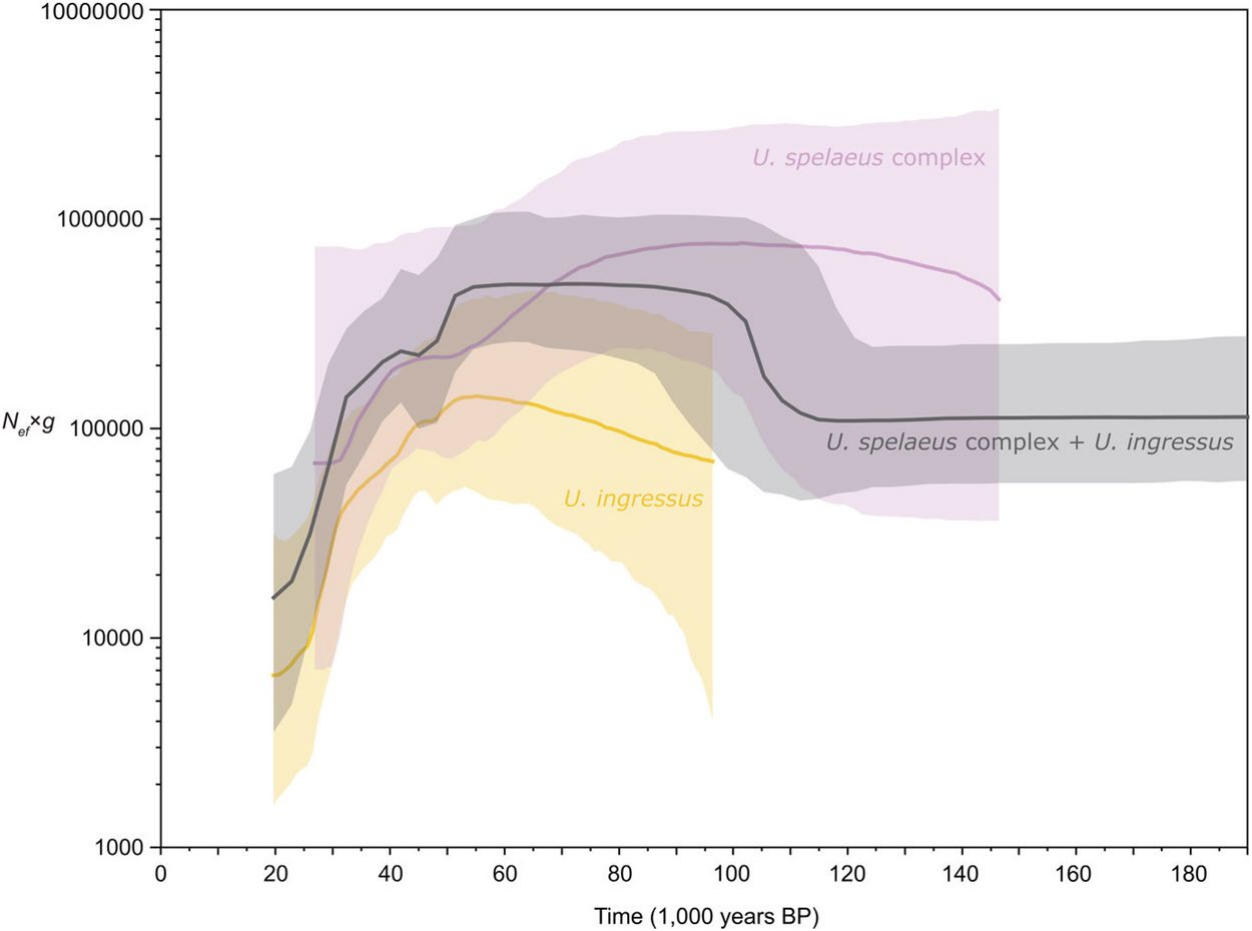
Bayesian skyline plot derived from an alignment of 123 complete mitochondrial genomes. Effective female population sizes (*N*_*ef*_) times generation time (*g*) of all European cave bears (black), *Ursus ingressus* (orange), and the *Ursus spelaeus* complex (violet). x axis: time in ^14^C years before present; y axis: female *N*_*ef*_ x *g*; centre line: median *N*_*ef*_ x *g*; upper and lower bounds: limits of 95% highest posterior density intervals.

We calculated the changes in the effective female population sizes (*N*_*ef*_) of European cave bears during the Middle and Late Pleistocene and visualized them in Bayesian skyline plots (Fig. 3). In general, our population size reconstruction resembles previously published calculations, featuring a stable population size through the last 200 to 50 ka. This is intriguing, since this period of time encompasses two cold periods (MIS 6 and MIS 4), as well as two warmer periods (MIS 5 and the onset of MIS 3). However, our extended plot illustrates that the known initial reduction in population size starting about 50 ka BP is followed by a more drastic decline beginning 10 ka later and persisting until the ultimate extinction of the cave bear in Europe approximately 19 ^14^C ka BP (23 cal. ka BP) (Fig. 3).

## Discussion

### Phylogeography and population dynamics

Until now, the population dynamics of the European cave bear was mainly described by the westward migration of *U*. *ingressus* from South-Eastern Europe to the Eastern Alps starting 60 ka BP^45^. This event may be associated with a gradual increase in *U*. *ingressus N*_*ef*_, starting at the same time as the initial population decline of the *U*. *spelaeus* complex (Fig. 3). Subsequently, *U*. *ingressus* cohabited since 50 ka BP with the Alpine forms of the *U*. *spelaeus* complex, *U*. *s*. *ladinicus* and *U*. *s*. *eremus*, ultimately replacing these older haplotypes^42^ except in remote and/or high-altitude sites such as Bärenloch. Towards the onset of the LGM, the *U*. *ingressus* haplotypes retracted from their eastern habitats and advanced westwards, replacing the *U*. *s*. *spelaeus* haplotypes in the Ach valley at the eastern extension of the *U*. *s*. *spelaeus* distribution between 36 and 32 ka BP^12,29,42,46^.

Our results challenge this conclusion, considering that we were able to identify a deeply divergent branch from Casamène, France, clustering within the *U*. *ingressus* clade (Fig. 2, Figure S2). These specimens not only extend the *U*. *ingressus* distribution westwards across the Rhine river, but they also predate the hitherto known presence of *U*. *ingressus* in Western Europe by nearly 15 ka^46^. Furthermore, the obtained mitochondrial genomes are only closely related to one other specimen from Zoolithen cave, Germany^40^. Besides Herdengel, Austria, and the Ach valley caves in Southern Germany, Zoolithen cave represents a third site where both major haplogroups were genetically observed^40,42^. However, Zoolithen cave is the only of these three cases where the *U*. *s*. *spelaeus* remains are potentially younger than the *U*. *ingressus* fossil, previously molecularly dated to 37.7^42^ or 49^41^ ka BP. In comparison, the *U*. *s*. *spelaeus* specimens are dated to be younger than 34.2^42^ or 56.9^41^ ka BP according to Stiller^42^ respectively Fortes and colleagues^41^. In Casamène, *U*. *ingressus* and *U*. *s*. *ladinicus* haplotypes coexisted at least ~4 ka between 47.2 and 43.2 ka BP, whereby *U*. *ingressus* inhabited the cave from 49.3 to 34.5 ka BP. Since a basal *U*. *ingressus* lineage continuously populated parts of the French Jura already for 15,000 years before the Ach valley haplogroup replacement, it appears unlikely that the expansion of *U*. *ingressus* occurred as a single, gradual westward migration. Instead, our results suggest that the original distribution of *U*. *ingressus* spanned much larger parts of Central Europe than previously assumed and that some relict populations persisted after the eastwards shift of their habitat.

Our analyses revealed new evidence for a second, eastward migration of *U*. *ingressus*. Noticeably, 16 out of 17 Serbian samples branch within the South-Eastern *U*. *ingressus* subclade. However, one specimen from Vrelska clusters with samples that originate from the Ural Mountain region (Bolshoi Glukhoi, Medvezhiya and Serpievskaya caves) within the Central European *U*. *ingressus* subclade. This result may reflect, as previously suggested by Baca and colleagues^47^, eastward migrations or gene flow between Central Europe and the Ural Mountains populations. A similar geographical pattern of gene flow between the European aurochs and the Eurasian steppe bison was recently reported by Soubrier and colleagues^48^. However, so far, this hypothesis was only supported by the comparatively old samples from Niedźwiedzia cave, Poland, dating between 41.5 and 87 ka BP^47,49^. In comparison, no published date of *U*. *ingressus* remains from the Ural Mountain region is older than 47.6 ka BP so far^47,49^. The Polish specimens share mitochondrial control region haplotypes closely related to the Ural Mountains ones, potentially indicating long-distance genetic exchange^49^. Forming an outgroup to all three Russian sequences, our sample from Vrelska cave suggests that this migration or gene flow from Central Europe did not only comprise the East European Plain north of the Carpathian Mountains but also the Southern Balkan Peninsula and that the distribution of this Central European haplogroup at the time of its highest diversity^42^ extended further eastward at least until 44 ka BP, potentially representing a taxon previously designated as *Ursus kanivetz kanivetz* by Baryshnikov and colleagues based on morphological features^18,50^. In contrast, specimens from Serbia belonging to the South-Eastern group all together yield radiocarbon dates ranging between >49 and 33 ka BP. The persisting presence of only the Southern-Eastern lineage and the lack of the Central European one may be associated with a subsequent replacement of the latter population at the Southern Balkan Peninsula. However, more mitochondrial genomes from South-Eastern Europe are needed to confirm this hypothesis. Focusing on the North-Eastern Italian cave bears from Paina, Trene and Broion, these samples exhibit closest genetic affinities to other Central European *U*. *ingressus* individuals from Austria, Slovenia and Southern Germany. Interestingly, all four radiocarbon dated specimens younger than 25 ka BP (TU841, TU843, TU844 and TU855) form together a monophyletic clade, suggesting low genetic diversity within the relict population of the Berici Hills.

In an analogous manner, we investigated the phylogeography of the *U*. *spelaeus* complex. To infer the topology within the *U*. *spelaeus* clade, we generated sequences of *U*. *s*. *eremus* from Bärenloch as well as the first mitogenome of *U*. *s*. *ladinicus* from Casamène and Prélétang. Notably, it was not possible to reliably phylogenetically classify the cave bears from Bärenloch using the mitochondrial control region-based approach. While these phylogenetical reconstructions place the Bärenloch specimens within the *U*. *s*. *ladinicus* branch (Figure S1, Figure S3), the tree featuring complete mitochondrial genomes yields another topology (Fig. 2, Figure S1, Figure S2). These findings confirm that phylogenetic relationships within branches of the mitochondrial control region tree should be interpreted with caution as discussed previously^44^. This observation could be explained by the impact of recurrent mutation that appears most acute in closely related control region haplotypes exhibiting a low level of evolutionary divergence as proposed by Knaus and colleagues^44^. Using complete mitochondrial genomes, *U*. *s*. *eremus* forms a distinct outgroup to all the other *U*. *spelaeus* sequences. Regarding *U*. *s*. *ladinicus*, our analysis demonstrated that this group previously described as monophyletic^45^ forms a paraphyletic group branching off basally to the *U*. *s*. *spelaeus* clade, and comprising haplotypes from Casamène, Prélétang, and the Grotte d’Ours. Thus, the control region haplogroup previously labelled as *U*. *s*. *ladinicus*^19^ does not represent a genetically distinct unit (based on the mtDNA), but rather a transitional form between the MRCA shared with *U*. *s*. *eremus* and the typical *U*. *s*. *spelaeus* haplotypes. This again emphasizes that previous taxonomic classifications based on morphology are not fully congruent with mtDNA haplogroups^20,21,29^. However, without knowledge of the nuclear genome, it is not possible to exclude gene flow as a potential reason for shared haplotypes as indicated by recent analyses of the autosomal cave bear genome^21^. In general, the basal position of these specimens as well as of *U*. *s*. *spelaeus* samples from Zoolithen cave, Germany, within the *U*. *spelaeus* haplogroup may indicate an Eastern French or North-Western alpine origin of the *ladinicus/spelaeus* (sensu stricto) haplogroup complex.

### Extinction

As demonstrated by Stiller and colleagues^11^, the start of the cave bear population decline preceded its final extinction by approximately 25 ka. A slow and continuous decline may be correlated with changing environmental conditions. Yet, climate and associated vegetation change as main factors appear improbable, albeit their strictly herbivorous feeding preferences remained unchanged during the Late Pleistocene^12,24,51^, since cave bears were well adapted to severe climate as indicated by their appearance beyond the Arctic Circle^16^. This is also congruent with our estimations of the cave bear population size development through the last 150 to 25 ka, exhibiting a relatively stable population size even during the two cold periods MIS 6 and MIS 4. Also, the multiple Heinrich cooling events during MIS 3 did apparently not coincide with sudden decreases in population size. Therefore, it appears unlikely that these previous climatic fluctuations did substantially affect the cave bear population in Europe. Furthermore, as emphasized by Stiller and colleagues^11^, the cooling climate of the beginning LGM did not start before 30 ka BP, nearly 20 ka after the beginning of the cave bear population decline, suggesting instead a major impact of human activities related to the expansion of modern humans in Europe that took place at the same time^52–54^. As documented in the present study, the cave bear demise did not proceed slowly. Although the initial decline in population size started shortly before 50 ka BP during the end of the Mousterian associated with Neanderthals, the more drastic downturn of the European cave bear took place at around 35 to 40 ka BP at the onset of the Aurignacian and the expansion of anatomically modern humans in Europe^55^. As demonstrated by Fortes and colleagues^41^, cave bears supposedly exhibited a homing behaviour indicated by the strong association between mitochondrial haplotype and cave. Such high dependence of cave bears on their birth caves may have created severe competition with Neanderthals, but especially with anatomically modern humans^41^. This was due not only to growing human density and group sizes but also increased human residence times^11,35,36,52^. These factors as well as the introduction of new technology (such as simple and split-based bone points) to more efficiently extract animal nutritional resources^52,56^, made the cave bears also more at risk for direct hunting by hominins^32,36–38,57^. Especially for North-Eastern Italy, namely Rio Secco Cave, Fumane Cave and the Berici Hills sites (Paina and Trene), persistence of cave bear exploitation from the Late Neanderthal to the anatomically modern human occupation during the last 50 ka years was recently reported^24,32,38^, supporting hunting evidence from other European locations such as the Ach valley caves^36^, Germany, or Potočka zijalka^37^, Slovenia. The negative human effect on cave bear populations would have been increased at the onset of the Last Glacial Maximum by the cooling climate and subsequently lower vegetation productivity, fragmenting the population into various subpopulations inhabiting small refugial habitats^12^ with suitable, stable microclimates and thus a broad range of available plant types (such as the Berici Hills^24^) as suggested by Baca and colleagues^23^. For many late Pleistocene megafauna species, such reductions in habitat range, population size and genetic diversity are intrinsically linked over evolutionary time^3^. This seems also congruent with results reported by Cooper and colleagues, indicating that humans canalised cave bear metapopulation extinction by interrupting the subpopulation connectivity^7^. Until the end of the maximum extent of the Scandinavian Ice sheet, only a few isolated populations survived across Central and Eastern Europe^22–24^. Consequently, the *U*. *ingressus* specimens from Stajnia cave and the Venetian Pre-Alps (Paina and Trene) dating to 26 and 25 cal. yr. BP^23^ and between 24 and 23 cal. yr. BP^24^ respectively represent the genetically impoverished relict of the much larger and more diverse cave bear population in Europe.

Thus, our study highlights the potential role of human activity in the general extinction and local extirpation of the European cave bear. Furthermore, we have shown that our current knowledge of cave bear phylogeography is biased by the employed methodology and the small and/or sparse number of sampled specimens. Using complete mitochondrial genomes covering spatially widespread sites can mitigate these problems, allowing such studies to gain deeper insight into the population dynamics of Late Pleistocene megafauna species such as the cave bear. Even if these insights are restricted to the maternal lineage and limited by the available number and length of DNA sequences, the conclusions already provide a more detailed understanding of cave bear population dynamics than previous studies. In the future, new high quality AMS radiocarbon dates of more extensive geographic coverage and, in particular, nuclear DNA data, combined with high-resolution palaeoecological data for each population and individual studied, will shed light on the evolution and extinction of cave bears.

## Material and Methods

### Sample collection

We selected 81 assumed cave bear specimens to be included in this study, featuring geographic regions that have been underrepresented in previous genetic work. We then generated 59 complete mitochondrial genome sequences using the approach described below. In addition, we obtained direct Accelerator Mass Spectrometry (AMS) radiocarbon dates on bone collagen for 24 samples for which no ^14^C age was available so far.

### DNA extraction

To minimize environmental contamination, bone samples were exposed to UV-light at least 30 minutes from all sides. Afterwards, 30–54.9 mg bone powder was removed from the inner substantia compacta of a long bone of each specimen using a dentistry drill. Afterwards, ancient DNA was extracted according to the method described by Dabney and colleagues^14^. DNA extracts were converted into double-indexed Illumina libraries using the approaches described elsewhere^58,59^. All extractions and pre-amplification steps of the library preparation were performed in a designated ancient DNA clean room facility. Indexed libraries were amplified in 100 μl reactions with AccuPrime *Pfx* and Herculase II Fusion followed by purification. Target enrichment of mitochondrial DNA was performed by in-solution capture of the pooled libraries using baits generated from modern polar bear (*Ursus maritimus*) mitochondrial DNA as described by Furtwängler and colleagues^60^. Finally, the enriched libraries were multiplex sequenced on an Illumina HiSeq. 4000 using 75 + 8 + 8 cycles at the Max Planck Institute for Science of Human History, Jena, Germany.

### Sequence processing

De-indexing was performed by sorting all sequences corresponding to their p7 and p5 index combinations. Next, read processing, including adaptor trimming, quality filtering and duplicate removal, was performed using the software EAGER^61^. Mapping of single-end reads to a reference cave bear mitochondrial genome (NC_011112.1) using CircularMapper and generating 3-fold consensus sequences was also conducted using EAGER^61^. To control for damage-derived substitutions, we applied an established set of criteria^62,63^ to create the consensus sequences and alignments: a minimum non-duplicate coverage for a position to be called (3X) and a minimal allele frequency for a call to made (75%). When either are not met an ‘N’ is called. Only sequences for which no more than 30% of bases are called as N (minimum 70% 3X coverage) are included in the alignment. Additionally, a second alignment using a stricter set of criteria^62,63^ (minimum 10X non-duplicate coverage for a position to be called, minimal allele frequency 90%, minimum 80% 10X coverage) was generated to examine possible incongruities between the tree topologies caused by low-coverage samples.

### Data from other studies

We included in our study complete mitochondrial genome sequences of 64 European^40,41,64^ and 2 Caucasus cave bears^40^ previously published. For Bayesian analyses, *Ursus kudarensis* cave bears from Hovk^40^, Armenia, were subsequently excluded.

### Alignment and model selection

Multiple Sequence Alignment was conducted in MAFFT 7.310^65,66^. Model selection was performed using ModelFinder, integrated in IQ-Tree 1.5.5^67^.

### Inferring phylogenetic relationships

Phylogenies were constructed from a total of 16,360 positions using MEGA 7.1.014^68^ and IQ-Tree 1.5.5 including ultrafast bootstrap^69^. Maximum-likelihood topologies were generated for all positions for which coverage was at least three-fold in each of the reconstructed sequences. Alignment columns with gaps or missing data were included. Bootstrap support values were obtained over 10,000 replicate data sets, using the American black bear (*Ursus americanus*, JX196366.1) as an outgroup. The phylogenetic trees were edited in FigTree version 1.4.3 (http://tree.bio.ed.ac.uk/software/figtree).

### Comparison of D-Loop and Mitogenome Tree Topology Differences

To identify D-Loop coordinates within our reconstructed mitochondrial sequences, these were aligned to D-Loop sequences of *Ursus spelaeus* previously published by Stiller and colleagues^42^ using MEGA 7.1.0 and sequences outside of the aligned regions were discarded. Calculated maximum-likelihood topologies were then compared using the tanglegram function integrated in Dendroscope 3.5.9 by Huson and Scornavacca (http://dendroscope.org)^70^.

### Bayesian phylogenetic inference and demographic reconstruction

Dated Bayesian phylogeny and demographic reconstructions were obtained using BEAST 1.8.4^71^. The alignment, including 59 new mitogenomes and 64 previously published mitogenomes, 16,360 nt long, was partitioned using PartitionFinder 2.1.1^72^ using six input data blocks (noncoding, tRNA, rRNA, and codon position 1, 2 and 3 of the protein coding genes), greedy search scheme and BIC model selection. The selected five partitions (tRNA and rRNA were combined into one partition by PartitionFinder) were included in BEAST analysis. We used tipdating to calibrate the relaxed molecular clock (uncorrelated, lognormal, separate clock for each partition). For radiocarbon and stratigraphy-based dated samples we used midpoint ages as point tipdates. For samples, for which age estimates have been previously estimated using molecular dating and are published, we used their ages as distributions rather than points in the analysis with normal priors (the estimated molecular age as the mean and 10% of the molecular age estimate standard deviation). Undated samples were assigned uniform age priors between 20,000 and 120,000 years old. Results of the molecular dating of these samples (median estimated age plus 95% credibility intervals as well as posterior density distribution for each estimate) can be found in Table S1 and Figure S4. Bayesian skyline population model was used. Markov Chain was run under for 200 million steps with sampling every 20,000^th^ step. Mixing and convergence was inspected using Tracer 1.7.1^73^.

### Accession numbers

The demultiplexed sequencing data for the 59 cave bear mtDNA genomes is deposited in the NCBI SRA Archive with the BioProject ID PRJNA545596.

## Supplementary information

Supplementary Info
