# Supplementary material for: Large-scale mitogenomic analysis of the phylogeography of the Late Pleistocene cave bear
Source: Sci Rep. 2019 Aug 15;9:10700. doi: 10.1038/s41598-019-47073-z (PMC6695494; doi:10.1038/s41598-019-47073-z)
Supplement: Supplementary file 1 — Supplementary Info [file 41598_2019_47073_MOESM1_ESM.pdf]

Large-scale mitogenomic analysis of the phylogeography of the Late Pleistocene cave bear

Joscha Gretzinger[1,2], Martyna Molak[3], Ella Reiter[1], Saskia Pfrengle[1], Christian Urban[1,4], Judith Neukamm[1,4], Michel Blant[6], Nicholas J. Conard[7,8], Christophe Cupillard[9], Vesna Dimitrijević[10], Dorothée G. Drucker[8], Emilia Hofman-Kamińska[11], Rafał Kowalczyk[11], Maciej T. Krajcarz[12], Magdalena Krajcarz[13], Susanne C. Münzel[1], Marco Peresani[14], Matteo Romandini[14,15], Isaac Rufí[16], Joaquim Soler[16], Gabriele Terlato[14], Johannes Krause[1,2], Hervé Bocherens[8,17]*, Verena J. Schuenemann[1,4]*. (*corresponding authors)

1. Institute for Archaeological Sciences, University of Tübingen, Tübingen, Germany
2. Max Planck Institute for the Science of Human History, Jena, Germany
3. Museum and Institute of Zoology, Polish Academy of Sciences, Warsaw, Poland
4. Institute of Evolutionary Medicine, University of Zürich, Zürich, Switzerland
6. Swiss institute for speleology and karst studies (SISKA), La Chaux-de-Fonds, Switzerland
7. Ancient prehistory and quaternary Ecology, University of Tübingen, Tübingen, Germany
8. Senckenberg Centre for Human Evolution and Palaeoenvironment (S-HEP), University of Tübingen, Tübingen, Germany
9. Service Régional de l'Archéologie de Bourgogne-Franche-Comté and Laboratoire Chronoenvironemment, CNRS, UMR 6249, Besançon, France
10. Laboratory for Bioarchaeology, Department of Archaeology, University of Belgrade, Belgrade, Serbia
11. Mammal Research Institute, Polish Academy of Sciences, Białowieża, Poland
12. Institute of Geological Sciences, Polish Academy of Sciences, Warsaw, Poland
13. Institute of Archaeology, Nicolaus Copernicus University in Toruń, Toruń, Poland
14. Department of humanities, Section of Prehistoric and Anthropological Sciences, University of Ferrara, Ferrara, Italy
15. Department of Cultural Heritage, University of Bologna, Ravenna, Italy
16. Institute of historical research, University of Girona, Girona, Spain
17. Department of Geosciences, University of Tübingen, Tübingen, Germany

Supplementary Section 1 Archaeological sites with cave bears included in the present study

Bärenloch (Switzerland) (Michel Blant)

Cave bear (*Ursus spelaeus*) is the dominant species in Pleistocene bones deposits of the Bärenloch cave in Préalpes fribourgeoises (Charmey, altitude 1645 m a.s.l., 46° 38' N 7° 16' E). Bones are dated between 47 to 28 ka cal. BP. They were discovered both at the entrance and inside the cave. The Bärenloch cave was certainly a hibernation and a birthing den for cave bears. Cave lion, brown bear, wolf, ibex and others were also identified in the Pleistocene fauna of the site. The cave bear population of Bärenloch became extinct during the cooling period at 27.8 ka cal. BP, preceding the Last Glacial Maximum (LGM)[1].

Casamène (France) (Christophe Cupillard)

Casamène cave is located at the western part of the Jura range, in the Doubs valley, at 383 m a.s.l. Discovered in 1912 as a paleontological site, the cavity has been partially excavated by Pierre Pétrequin and his team between 1968 and 1970. This archaeological excavation was conducted on a surface of 45m^2 at the entrance of the cave and yielded a 4 m thick deposit subdivided in 13 layers (layer 0 to layer XII). The layer I provided few Upper Palaeolithic artefacts, whereas in the levels Ic, II, Vb, Vc, VIb, VII, VIII, IX and X, there are less than an hundred lithic Mousterian artefacts and a fauna dominated by *Ursus spelaeus* according to the study of François Prat (University of Bordeaux I) who also recognized *Ursus arctos, Cervus elaphus, Capra ibex, Rupicapra rupicapra, Sus scrofa, Panthera pardus, Crocuta crocuta, Canis lupus, Vulpes vulpes, Castor fiber and Marmota marmota*[2]. Since 2013, the study of this site has been re-evaluated in the frame of a new archaeological research program led par C.

Cupillard[3] and, for the first time, the archaeological layers have been radiocarbon dated with animal bone carefully determined and selected by S. C. Münzel. From the layers I to VI, 9 radiocarbon dates have been directly obtained from cave bear bones, 6 of which are presented in this paper, and range from 30,518 ^{14}C yr. BP to 47,406 ^{14}C yr. BP.

Hohle Fels (Germany) (Nicholas J. Conard, Susanne C. Münzel)

Hohle Fels is a cave site in the Ach Valley (Swabian Jura) between Blaubeuren and Schelklingen. The cave has a long research history and was first recognized by findings of cave bear bones in the middle of the 19th century. Initial systematic excavations were undertaken by Oscar Fraas 1870/71, a palaeontologist of the Königliche Naturalienkabinett in Stuttgart. Modern excavations were started by the University of Tübingen in 1977-1979 and 1987-1996 by Joachim Hahn, which were continued by Nicholas Conard since 1997 with yearly seasons until now. Hohle Fels cave is one of six cave sites in the Swabian Jura, namely Geißenklösterle, Sirgenstein (Ach Valley), Vogelherd, Bockstein and Hohlenstein Stadel (Lone Valley), which recently were inscribed in the list of UNESCO World Heritage sites for its oldest musical instruments and oldest figurative art[4,5]. Beside these outstanding finds of Palaeolithic flutes and ivory figurines, cave bear research was an important focus especially in Hohle Fels. Here, the first irrefutable proof of cave bear hunting was found by a thoracic vertebra with an embedded flint projectile in the processus transversus[6]. The vertebra was found in one of the Gravettian layers and is dated to 27 830+150-140 BP (KIA-17743). This together with an increasing number of cut and impact marks, as well as other modifications, from the Middle Palaeolithic to the Aurignacian and Gravettian layers proves consistent hunting of this species and increasing impact on the cave bear population found in Hohle Fels[7].

Prélétang (France) (Hervé Bocherens)

The cave of Prélétang is located in the Vercors massif, SW of the town of Grenoble in France, at an altitude of 1225 m a.s.l.[8,9]. The cave is formed in Late Cretaceous (Urgonian) limestone. This site yielded numerous cave bear remains as well as Mousterian lithic artefacts. The fossil material studied here is from excavations led by Thierry Tillet from 1994 to 1999 in the entrance area of the cave[9]. All cave bears analysed from this site exhibit collagen $\delta^{13}C$ and $\delta^{15}N$ values indicative of a vegetarian diet[10].

L'Arbreda (Spain) (Isaac Rufí, Joaquim Soler)

Arbreda cave is located in the municipality of Serinyà, in NE Catalonia (Spain), in the interface between middle range mountains of the Catalan Transversal Range and the Banyoles lacustrine system, at around 200 meters over sea level. The Arbreda cave and its neighbouring Palaeolithic caves of Mollet, Mollet III, Pau, Roure and Reclau Viver are all found along the same 200 meters long travertine cliff (Reclau's Place), which allows to track the natural and cultural evolution in the same place of the Western Mediterranean since the Middle Pleistocene. In particular, Arbreda cave preserves a very good record of the classical Mousterian (120,000 – 40,000 BP), Archaic Aurignacian, Evolved Aurignacian, Gravettian, Middle Solutrean and Upper Solutrean periods, with a minor presence of the Magdalenian and Neolithic phases as well[11]. Concerning the *Ursus spelaeus* record, which represents the 47% of the determined faunal remains in whole Middle Palaeolithic levels, it clearly and abruptly vanishes at the beginning of the Upper Palaeolithic, during the Archaic Aurignacian[12]. The samples from Arbreda cave included in this paper come from the Mousterian level I, which has an age determined by an ensemble of ultrafiltrated ^{14}C dates on bones resulting in: 32,100±450 yr. BP (OxA-21663), 32,300±450 yr. BP (OxA-21703), 37,300±800 yr. BP (OxA-21662), 39,200±1,000 yr. BP (OxA-21704) and 44,400±1,900 yr. BP (OxA-21702)[13]. There is still

another ultrafiltrated date obtained from a charcoal which yielded a result of 38,350±400 yr. BP (OxA-19994)[14].

Perspektywiczna Cave (Poland) (Maciej T. Krajcarz, Magdalena Krajcarz)

Perspektywiczna cave is located in southern Poland, in the middle part of Kraków-Częstochowa Upland (N 50°26'34.0" E 19°46'01.0"). The entrance to the cave is situated at 345 m a.s.l., at the base of a limestone cliff, with western exposition. The cave has been continuously excavated since it was discovered in 2012 and is still under excavation. Research revealed the presence of at least two chambers containing stratigraphic series of Holocene and Upper Pleistocene sediments rich in animal bones and traces of human settlement. Stratigraphy of the site is complicated due to the varied morphology of the bedrock and post-depositional colluvial disturbances. 20 lithological layers with >4 m total thickness were identified in the sedimentary fill of the lower chamber, and further 4 layers with 2.5 m thickness in the upper chamber. The dating of the sediments is based on various methods: archaeological (materials from late Paleolithic to modern[15]), biostratigraphic (faunistic assemblages typical for the Last Glaciation, Late Glacial and Holocene up to contemporary fauna[15]), lithostratigraphy (lithostratigraphic units VI and VIII-IX of the scheme by Madeyska 1988[16], and unit E of cave loess according to Krajcarz et al. 2016[17]) and chronometric dating (so far, a series of 76 radiocarbon dates and four thermoluminescent dates has been obtained[18]). Most of the cave bear remains were found in one sedimentary series in the upper chamber dated to 30,000-50,000 cal. BP, and some in the colluvial sediments re-deposited to the lower chamber. All cave bear bones were directly dated with a use of radiocarbon method obtaining the age of 40,200±1,200 to 47,538±1337 ^{14}C yr. BP (around 43,000 to >49,000 cal. yr. BP). The faunal assemblage associated with cave bears shows a dominance of cave-dwelling carnivorans such as cave bears and cave hyenas, with lower number of ungulates: *Rangifer tarandus*, *Coelodonta*

antiquitatis, *Bos/Bison* and *Megaloceros giganteus*. No archaeological record was associated with cave bear strata in the cave, but its chronology is contemporaneous with late Middle Paleolithic and Jerzmanowician settlement in the region[19].

Paina (Italy) (Marco Peresani, Matteo Romandini, Gabriele Terlato)

Paina cave is located in the Berici Hills in northeastern Italy, about 350 m a.s.l., on the edge of a steep slope connecting the plateau to the alluvial plain. Field investigations revealed a stratigraphic series of about 1.50 m in thickness, which includes 12 layers containing Middle (Units 12–10) and Upper Palaeolithic (Units 9–5) artefacts. The focus is the zooarchaeological content of layers 5 and 6, radiocarbon dated from 20,120 ± 220 to 19,430 ± 150 ^{14}C yr. BP (layer 6) and to 19,861 ± 70 ^{14}C yr. BP (layer 5) based on cave bear bones[20,21]. The lithic artefacts have been referred to the Early Epigravettian with shouldered points. The faunal remains of these units record the predominance of carnivorans (cave bear, fox and mustelids) over ungulates. Amongst the latter, the most abundant species are the cervids (*Cervus elaphus* and *Alces alces*), followed by caprids and wild boar[21,22].

Buso doppio del Broion (Italy) (Matteo Romandini, Gabriele Terlato)

The Buso doppio del Broion Cave is a karst cavity of Berici Hills (North-Eastern Italy), opening at 150 m a.s.l. on their eastern slope. The cave contains Upper Pleistocene deposits and the archaeological excavation is ongoing, carried out by University of Ferrara. It is formed by a system of galleries that reaches a horizontal depth of more than 17m. The stratigraphic sequence provides evidence of short-term human occupations at around the end of MIS 3 and the onset of the LGM. The finding of several lithic implements (among which a few shouldered points) mostly in the upper reworked sediment, suggests early Epigravettian frequentation, whereas the presence of gravettes and microgravettes in the medium-basal portion of Layer 1

is indicative of Gravettian[23]. The lower portion of the stratigraphic sequence (Layers 4-7) provided other lithic implements (micro-bladelets, possibly Aurignacian), the study of which is still underway[22,23]. The focus is the zooarchaeological content of Layers 1 and 2. The faunal remains of these layers record the predominance of carnivores (*Ursus spelaeus*, *Vulpes vulpes*, *Felis silvestris*, *Canis lupus*) over ungulates (*Alces alces*, *Cervus elaphus*, *Rupicapra rupicapra*). The sediments also contained fish remains, which mostly consist in cyprinid and salmonid vertebrae, and birds[23,24]. Human modifications such as cut-marks are present on ungulate and cave bear bones. The amount and state of preservation of cave bear remains suggest that this mammal used the cave as a shelter for several hibernation cycles, comparably to other caves in Berici Hills.

Trene (Italy) (Marco Peresani, Matteo Romandini, Gabriele Terlato)

Trene cave is located in the Berici Hills in north-eastern Italy, at about 360 m a.s.l. The cavity was the subject of systematic excavations done by the University of Ferrara in 1956, which yielded a 1.14 m thick deposit, subdivided into three macro-units: A, B and C. The focus of this paper is the macro-unit B with radiocarbon dates on ungulate bones ranging from 17,640 ± 140 ^{14}C yr. BP to 18,630 ± 150 ^{14}C yr. BP. The lithic industry ascribes the anthropic frequentation to the early Epigravettian. The faunal assemblage shows a clear dominance of cave bear over other taxa, followed by ungulates (*Alces alces*, *Cervus elaphus* and *Sus scrofa*), fishes and birds[24].

Vrelska Cave (Serbia) (Rafał Kowalczyk)

Vrelska cave is located in the town of Bela Palanka, some 20 m above the spring, at the altitude of 545 m. The entrance is 2.3 m wide, 1.8 m high, and was completely closed before the interventions in purpose of exploiting the spring. The total length of the cave is 68 m[25].

Paleontological excavations were carried out in 1990. Numerous remains of vertebrates were found, small mammals in particular (*Sorex araneus*, *Crocidura leucodon*, *Rhinolophus hipposideros*, *Myotis myotis*, *Lepus sp.*, *Ochotona pusilla*, *Spermophilus citellus*, *Sicista subtilis*, *Nannospalax leucodon*, *Glis glis*, *Apodemus sylvaticus*, *Cricetus cricetus*, *Mesocricetus newtoni*, *Cricetulus migratorius*, *Clethrionomys glareolus*, *Arvicola terrestris*, *Chionomys nivalis*, *M. arvalis/agrestis*, *Terricola subterraneus*, *Lagurus lagurus*, *Canis lupus*, *Vulpes vulpes*, *Ursus spelaeus*, *Lynx pardina*, *Equus ferrus*, *Capreolus capreolus*, *Bos/Bison*[26].

Kovačevića (Serbia) (Rafał Kowalczyk)

The cave is located in the village Cerova, northeast of the city Krupanj in Western Serbia. The entrance to the cave is 29 m above the river Kovačevića reka, at the altitude of 495 m. The length of the cave is 985 m[27]. Cave bear remains have been collected during a reconnaissance conducted in 1985[26].

Vasiljska (Serbia) (Vesna Dimitrijević)

The cave is situated in Eastern Serbia, on southwest margin of the Knjaževac depression, in the canyon of the river Glodje cut in limestone slope of Devica mountain, some 80 m above the riverbed, at the altitude of 560 m. It is approximately 170 m long[25]. A small entrance and narrow channel lead to three chambers in row that are filled with Quaternary deposits, while the floor of the chamber in the background of the cave is barren, but abundantly ornamented with speleothems, especially massive stalagmites.

In the course of the reconnaissance in 1991, remains of Pleistocene mammals at the surface of three front chambers were collected, while small vertebrate remains were washed out from the section made by looters. Following mammal taxa were identified: *Talpa europaea, Sorex minutus, Crocidura leucodon, Rhinolophus ferrumequinum, Rh. euryale, Lepus* sp., *Ochotona*

pusilla, Sciurus vulgaris, Glis glis, Muscardinus avellanarius, Dryomys nitedula, Apodemus sylvaticus, Mesocricetus newtoni, Cricetulus migratorius, Clethrionomys glareolus, Arvicola terrestris, Terricola subterraneus, Microtus arvalis/agrestis, Chionomys nivalis, Canis lupus, Vulpes vulpes, Ursus spelaeus, U. arctos, Mustela nivalis, M. erminea, Felis silvestris, Capreolus capreolus, Capra ibex. Some remains of birds, reptiles and fish were also found.

Smolućka (Serbia) (Vesna Dimitrijević)

Smolućka cave is located in southwest Serbia, some 16 km southwest of Novi Pazar, in the Raška river basin. The entrance to the cave is about 15 m above the Smolućka river, at 945 m altitude. The total cave length is 25 m.

Archaeological excavations were performed from 1984 to 1987. In a section of Quaternary deposits 2.2 m deep, six layers were distinguished, without reaching the floor. More than two hundred flint artefacts were found, which by typological features are related to the Middle Palaeolithic – Mousterian techno-complex[28]. Mammal bone from the layer revealed AMS date of >38,000 years BP[29].

The faunal assemblage is taxonomically and palaeoecologically diverse, including 42 species of macro and micromammals: *Talpa europaea, Sorex araneus, S. minutus, Lepus* sp., *Ochotona pusilla, Spermophilus citellus, Glis glis, Muscardinus avellanarius, Dryomys nitedula, Sicista subtilis, Nannospalax leucodon, Apodemus sylvaticus, Mesocricetus newtoni, Cricetulus migratorius, Clethrionomys glareolus, Arvicola terrestris, Terricola subterraneus, Microtus arvalis, M. agrestis, Chionomys nivalis, Hystrix* sp., *Canis lupus, Vulpes vulpes, Ursus arctos, U. spelaeus, Mustela erminea, Mustela* sp., *Meles meles, Crocuta spelaea, Panthera pardus, Cervus elaphus, Megaloceros* giganteus, *Bos/Bison, Rupicapra rupicapra,* and *Capra ibex*[30]. In addition, 30 species of birds, 9 reptiles, 9 amphibians and 3 species of fish are identified[31]. Taphonomy of vertebrates' remains is complex, as some originate from cave dwellers while

majority is accumulated by mammal carnivores, bird of prey, and human hunters.

Mirilovska (Serbia) (Vesna Dimitrijević)

The cave is situated in the valley of the river Ravanica, right tributary of the Velika Morava river, 3 km upstream from the settlement of Senje. The entrance to the cave is approximately 70 m above the riverbed, at the altitude of 370 m. It is a karst cave in the form of a sub-horizontal channel, around 70 m long. Archaeological excavations were performed in 1995, up to the depth of 160 cm, within a small sondage (2 x 6 m). Six layers have been distinguished, with the top three dated to the Holocene age, the forth layer contained no faunal remains or any kind of artefacts, and the bottom two layers dated to the Pleistocene age. The few flint artefacts and single bone point are related to the Upper Palaeolithic[32].

Faunal remains originate from the cave dwellers, as well as from predator's prey. Cave bears were denning in the cave, mostly females with cubs, but occasionally also sole males. The cave was also home to red foxes and wolves. The remains of lagomorphs and rodents found in the cave most probably are brought in as red fox's prey. They are mostly represented by steppe species (*Ochotona pusilla, Cricetus cricetus, Mesocricetus newtoni*) whose living areas were at some distance from the cave. Finally, at certain periods man visited the cave, and remains of roe deer, chamois and ibex are probably remnants of his hunting.

Supplementary figures:

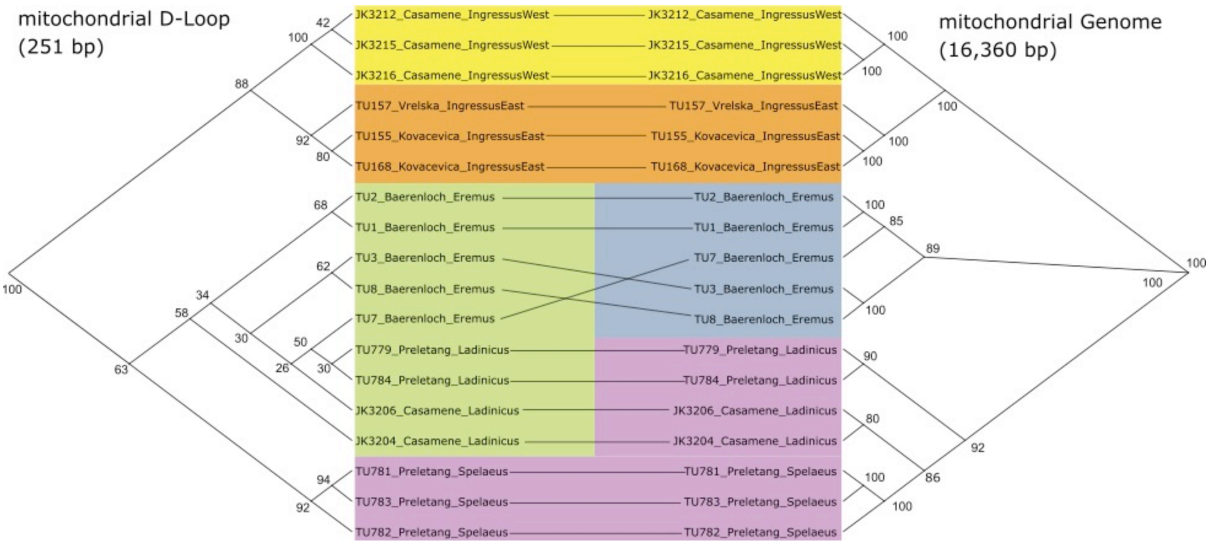

Supplementary Fig. 1: Tanglegram comparing mitochondrial genome and D-Loop sequence phylogenies.

Tanglegram comparing Maximum Likelihood phylogenies of 251 bp long D-Loop sequences (left) and complete mitochondrial genomes (right) for a set of 18 cave bear samples analysed in this study, featuring complete D-Loop sequences. Haplogroup clades based on the respective analyses are indicated by the colour coding matching Figure 1, *U. s. ladinicus* is marked in green.

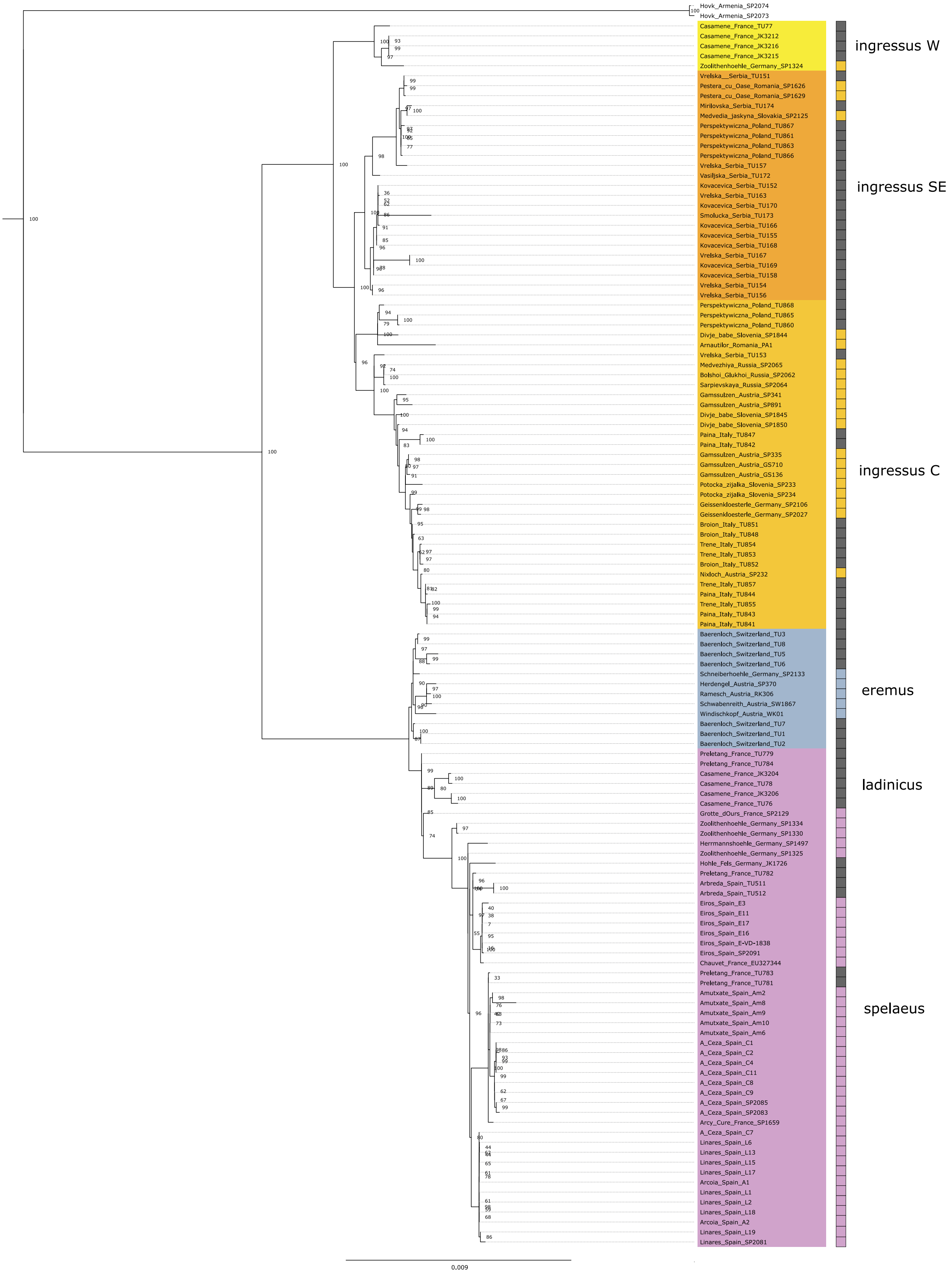

Supplementary Fig. 2: Maximum Likelihood tree constructed from a total of 16,360 positions of 125 European and Asian cave bear mtDNA sequences.

Bootstrap support values were obtained over 10,000 replicate data sets, using the American black bear as an outgroup. Haplogroup clades[33] are indicated by the colour coding matching Figure 1, *U. s. ladinicus* is marked in green. Morphology-based taxonomic identifications are represented as rectangular bars to the right of sample names.

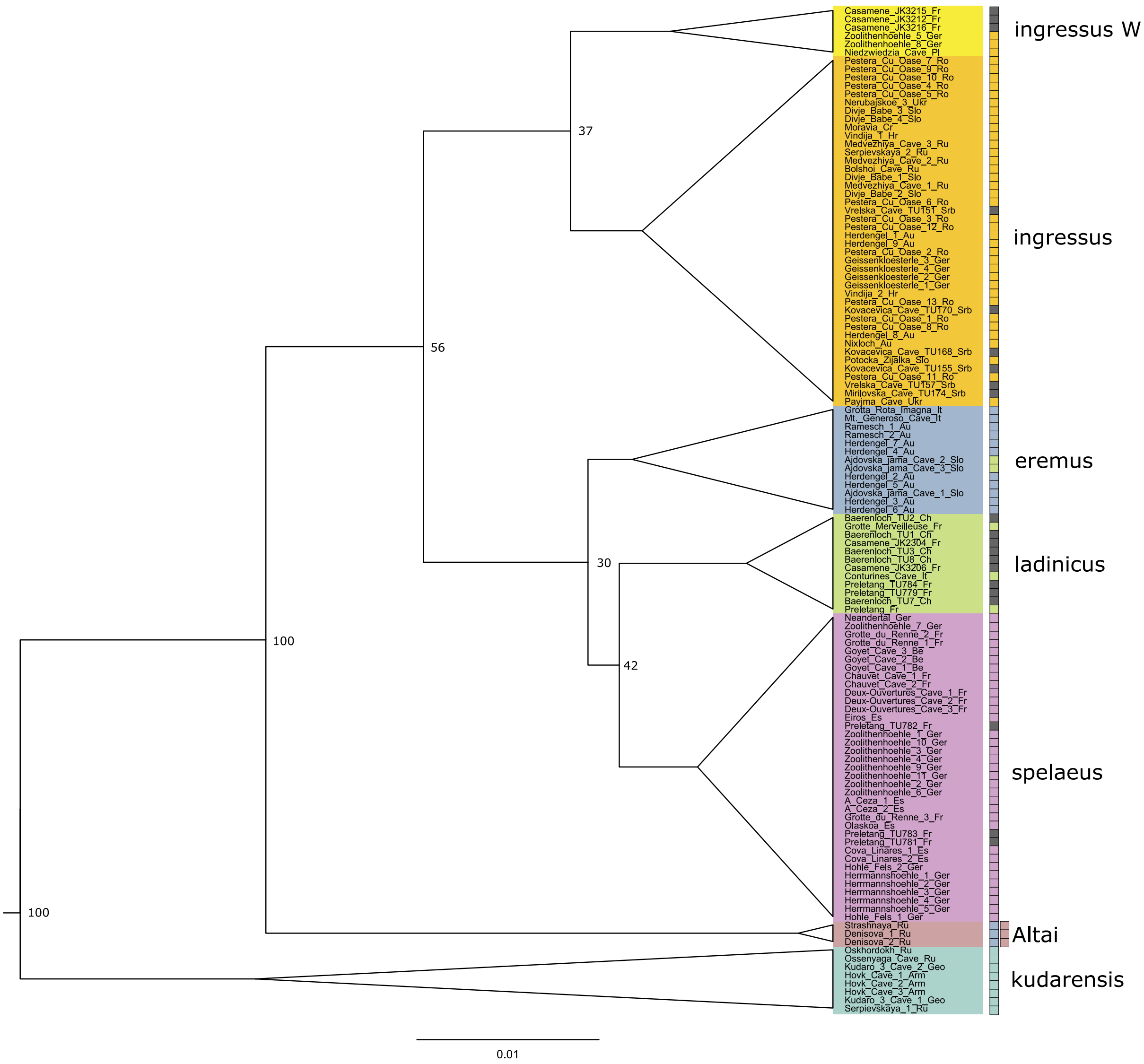

Supplementary Fig. 3: Maximum Likelihood tree constructed from a total of 251 positions of 121 European and Asian cave bear D-Loop sequences (including 21 new samples).

Bootstrap support values were obtained over 10,000 replicate data sets, using the European brown bear as an outgroup. The generally low Bootstrap values highlight the statistical

uncertainties that are inherent in analyses of the D-Loop. Haplogroup clades[33] are indicated by the colour coding matching Figure 1, *U. s. ladinicus* is marked in green. Haplogroup identifications[33] based on previous mtDNA analyses[34,35] are provided as rectangular bars to the right of sample names.

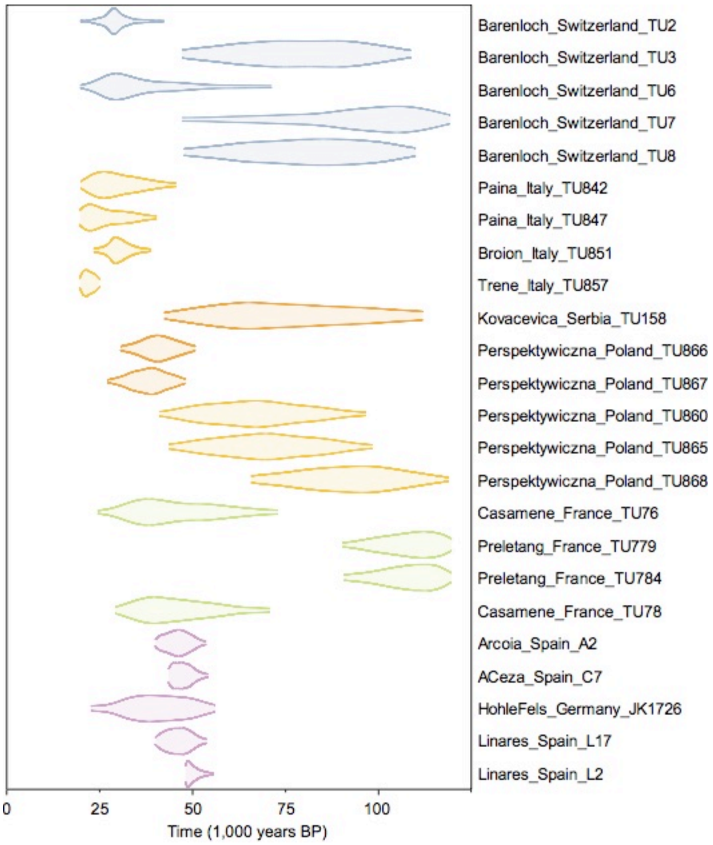

Supplementary Fig. 4: Posterior density distribution for each molecular age estimate (under a uniform 20-120 ka age prior) of previously undated samples.

Haplogroup clades[33] are indicated by the colour coding matching Supplementary Fig. 3.

Supplementary Table 1:

Median age and 95% credibility intervals for each molecular age estimate of previously undated samples.

Sample	Site	Classification	Median age	low 95% CI age	high 95% CI age
TU2	Bärenloch, CH	eremus	29,218	19,765	42,349
TU3	Bärenloch, CH	eremus	78,599	47,106	108,686
TU6	Bärenloch, CH	eremus	33,705	19,748	71,263
TU7	Bärenloch, CH	eremus	96,866	47,087	119,225
TU8	Bärenloch, CH	eremus	81,716	47,651	109,982
TU842	Paina, IT	ingressus C	29,240	19,808	45,541
TU847	Paina, IT	ingressus C	25,832	19,671	40,342
TU851	Broion, IT	ingressus C	30,005	23,418	38,695
TU857	Trene, IT	ingressus C	22,038	19,657	24,987
TU158	Kovacevica Cave, SRB	ingressus SE	74,451	42,345	111,892
TU860	Perspektywiczna Cave, PL	ingressus C	66,466	41,110	96,672
TU865	Perspektywiczna Cave, PL	ingressus C	69,745	43,571	98,274
TU866	Perspektywiczna Cave, PL	ingressus SE	40,767	30,637	50,723
TU867	Perspektywiczna Cave, PL	ingressus SE	38,412	27,003	48,082
TU868	Perspektywiczna Cave, PL	ingressus C	91,707	65,673	118,800
TU76	Casamène, FR	spelaeus	44,251	24,423	72,883
TU78	Casamène, FR	spelaeus	44,578	29,164	70,800
TU779	Prélétang, FR	spelaeus	108,368	90,301	119,653
TU784	Prélétang, FR	spelaeus	108,167	90,716	119,630
JK1726	Hohle Fels, GER	spelaeus	40,759	22,627	56,039
A2	Arcoia, ES	spelaeus	46,267	40,041	53,608

C7	A Ceza, ES	spelaeus	47,386	43,507	54,139
L2	Liñares, ES	spelaeus	49,777	48,006	55,620
L17	Liñares, ES	spelaeus	46,376	40,011	53,765

Supplementary Table 2:

Additional ^{14}C dating and collagen information for each sample sequenced in this study.

DNA ID	Archaeological ID	Dating ID	%C	%N	C/N	Age	SD
TU1	BRL-1	Ua-24794	39.8	14.0	3.3	28415	605
TU3	BRL-3	Ua-32591	42.5	14.0	3.5	40070	765
TU5	BRL-5	Ua-32591	40.9	14.0	3.4	26745	490
TU77	CSM_21	ETH-80732	41.2	14.2	3.4	47406	1309
JK3204	50 CSM_17	ETH-80731	41.0	15.1	3.2	39456	494
JK3206	46 CSM_19	ETH-51778	42.5	15.7	3.2	43890	999
JK3212	40 CSM_15	ETH-80729	41.4	15.4	3.1	38153	430
JK3215	41 CSM_16	ETH-80730	40.3	15.0	3.1	30518	170
JK3216	48 CSM_18	ETH-51777	40.9	15.2	3.1	41366	518
TU151	NHMB-8	ETH-80721	42.5	15.0	3.3	40470	567
TU152	NHMB-16	ETH-80725	42.6	15.3	3.3	48116	1432
TU153	NHMB-2	ETH-80715	38.2	13.3	3.4	40595	574
TU154	NHMB-3	ETH-80716	38.9	13.8	3.3	45918	1093
TU155	NHMB-19	ETH-80727	39.0	13.9	3.3	45673	1067
TU156	NHMB-6	ETH-80719	39.0	13.8	3.3	42687	740
TU157	NHMB-7	ETH-80720	42.0	14.8	3.3	38330	440
TU163	NHMB-4	ETH-80717	42.6	14.9	3.3	44748	947

TU166	NHMB-18	ETH-80726	41.3	14.5	3.3	46429	1167
TU167	NHMB-5	ETH-80718	42.0	14.7	3.3	43641	826
TU168	NHMB-10	ETH-80722	37.5	13.4	3.3	46376	1167
TU169	NHMB-12	ETH-80723	37.3	13.1	3.3	40848	590
TU170	NHMB-14	ETH-80724	40.1	14.3	3.3	45449	1035
TU172	SRBI-3	ETH-82478	34.7	12.3	3.3	43027	445
TU173	SRBI-4	ETH-82479	28.4	9.1	3.6	30649	113
TU174	SRBI-5	ETH-80728	39.1	13.6	3.4	28807	149
TU781	G 2700	ETH-82475	40	15	3.2	42400	409
TU782	G 2800	ETH-82476	41	15	3.2	40423	330
TU783	G 3500	ETH-82477	40	16	3.2	38742	277
TU784	G 1000	ETH-82474	41	15	3.2	49788	1006
TU841	CBV2	ETH-82963	37.6	14.7	3.0	20015	46
TU843	CBV8	ETH-82964	38.6	15.0	3.0	19914	45
TU844	CBV9	ETH-82965	40.7	15.7	3.0	19975	46
TU846	CBV18	ETH-82966	39.7	15.2	3.1	38661	219
TU848	CBV23	ETH-79367	39.6	15.1	3.1	29001	123
TU852	CBV33	ETH-82967	32.8	12.4	3.1	25978	70
TU853	CVB39	ETH-82968	38.8	14.8	3.1	25290	66
TU854	CVB40	ETH-82969	35.8	13.6	3.1	24755	63
TU855	CVB42	ETH-82970	39.7	15.1	3.1	19656	44
TU860	IP-5	ETH-90790	41.1	14.8	3.2	41446	638
TU861	IP-6	Poz-61114	42	16	3.2	40200	1200
TU863	IP-9	Poz-61115	40	14	3.2	41600	1400
TU865	IP-14	ETH-90791	41.6	14.5	3.2	47538	1337

Supplementary References

1. Blant, M., Bocherens, H., Bochud, M., Braillard, L., Constandache, M., & Jutzet, J. M. Le gisement à faune würmienne du Bärenloch (Préalpes fribourgeoises, Suisse). *Bulletin de la société fribourgeoise des sciences naturelles* 99, 149-170 (2010).
2. Pétrequin, P., Piningre J.-F. & Urlacher, J.-P. La grotte moustérienne de Casamène à Besançon. *Gallia-Préhistoire* 18, 359-399 (1975).
3. Cupillard, C., Bridault, A., Cailhol, D., Carquigny, N., Couchoud, I., Drucker, D. G., Krause, J., Münzel, S. C., Posth, C., Valentin, F. & Woodhead, J. Des derniers Néandertaliens aux premiers agriculteurs dans le massif du Jura et ses marges. Programme Collectif de Recherches, projet d'opération pluriannuelle 2012-2015, rapport 2014. Besançon Service Régional d'Archéologie de Bourgogne-Franche-Comté et Laboratoire Chronoenvironnement, UMR 6249 du CNRS, 92 (2014).
4. Conard, N. J. A female figurine from the basal Aurignacian of Hohle Fels Cave in southwestern Germany. *Nature* 459, 248-252. doi:10.1038/nature07995 (2009).
5. Conard, N. J., Malina, M. & Münzel, S. C. New flutes document the earliest musical tradition in southwestern Germany. *Nature* 460, 737-740, doi:10.1038/nature08169 (2009).
6. Münzel, S. C. & Conard, N. J. Cave Bear Hunting in the Hohle Fels, a Cave Site in the Ach Valley, Swabian Jura. *Revue de Paléobiologie* 23, 877–885 (2004).
7. Wojtal, P., Wilczyński, J., Nadachowski, A. & Münzel, S. C. Gravettian hunting and exploitation of bears in Central Europe. *Quaternary International* 359-360, 58–71, doi:10.1016/j.quaint.2014.10.017 (2015).
8. LeQuatre, P. La grotte de Prélétang (commune de Presles, Isère). Le repaire d'ours des cavernes et son industrie moustérienne. *Gallia-Préhistoire* 9, 1-83 (1966).

9. Tillet, T. Les Alpes et le Jura. Quaternaire et Préhistoire ancienne. SGF Collection Geosciences, Éditions scientifiques GB, Paris, 257 (2001).
10. Bocherens, H. Isotopic tracking of large carnivore palaeoecology in the mammoth steppe. *Quaternary Science Reviews* 117, 42-71 (2015).
11. Soler, J., Soler, N., Solés, A. & Niell, X. La Cueva de la Arbreda del Paleolítico medio al neolítico. In: (ed.) Sala, R. *Los cazadores recolectores del Pleistoceno y del Holoceno en Iberia y el estrecho de Gibraltar*, 266-276 (Universidad de Burgos and Fundación Atapuerca, 2014).
12. Maroto, J., Ramió, S., Solés, A. & Soler, N. La davallada de l'ós de les cavernes durant el plistocè superior. L'exemple del nord-est de Catalunya. *Cypsela* 13, 137-141 (2001).
13. Wood, R.E., Arrizabalaga, A., Camps, M., Fallon, S., Iriarte-Chiapusso, M.-J., Jones, R., Maroto, J., de la Rasilla, M., Santamaría, D., Soler, J., Soler, N., Villaluenga, A. & Higham T. F. G. The chronology of the earliest Upper Palaeolithic in northern Iberia: New insights from L'Arbreda, Labeko Koba and La Viña, *Journal of Human Evolution* 69, 91-109 (2014).
14. Maroto, J., Vaquero, M., Arrizabalaga, A., Baena, J., Baquedano, E., Jordá, J., Julià, R., Montes, R., Van Der Plicht, J., Rasines, P. & Wood, R. Current issues in late Middle Palaeolithic chronology: New assessments from Northern Iberia. *Quaternary International* 247, 15-25 (2012).
15. Sudoł, M., Krajcarz, M., & Krajcarz, M. T. Wyniki interdyscyplinarnych badań Jaskini Perspektywicznej (Wyżyna Częstochowska) w latach 2014-2016. In: (ed.) Urban, J. *Materiały 50*. Sympozjum Speleologicznego, Sekcja Speleologiczna PTP im. M. Kopernika, 145-146 (Kraków, 2016).

16. Madeyska, T. Osady jaskiń i schronisk Doliny Sąspowskiej. In: *Tło przyrodnicze osadnictwa pradziejowego*. Wydawnictwa Uniwersytetu Warszawskiego (eds. Jaskinie, C. W. & Sąspowskiej, D.), 77–173 (Warszawa, 1988).
17. Krajcarz, M. T., Cyrek, K., Krajcarz, M., Mroczek, P., Sudoł, M., Szymanek, M., Tomek, T., Madeyska, T. Loess in a cave – Lithostratigraphic and correlative value of loess and loess-like layers in caves from the Kraków-Częstochowa Upland (Poland). *Quaternary International* 399, 13–30, doi:10.1016/j.quaint.2015.08.069 (2016).
18. Krajcarz, M. T., Krajcarz, M. & Sudoł-Procyk, M. Chronostratigraphy of the sediments of Perspektywiczna Cave. In: (eds.) Czyżewski, Ł., Sudoł-Procyk, M. *Materiały 52*. Sympozjum Speleologicznego. Sekcja Speleologiczna PTP im. M. Kopernika, 57-58 (Toruń, 2018).
19. Krajcarz, M. T., Krajcarz, M., Ginter, B., Goslar, T. & Wojtal, P. Towards a chronology of the Jerzmanowician – a new series of radiocarbon dates from Nietoperzowa Cave (Poland). *Archaeometry* 60, 383–401, doi:10.1111/arcm.12311 (2018).
20. Parere, V., Gurioli, F. & Sala, B. Analisi di mortalità dell'orso delle caverne del Pleistocene superiore delle Grotta di Paina (Colli Berici, Vicenza): una tana invernale di svezzamento. Atti del 5° Convegno Nazionale di Archeozoologia (Rovereto, Edizione Osiride, 2006).
21. Gurioli, F., Parere, V. & Sala, B. La fauna del Pleistocene Superiore nella Grotta di Paina (Colli Berici, Vicenza). Atti del 5° Convegno Nazionale di Archeozoologia (Rovereto, Edizione Osiride, 2006).
22. Romandini, M. & Nannini, N., Epigravettians hunters in the territory of the bear of caves: the case of Covolo Fortificato di Trene (Vicenza, Italy). *Anthropologie* 116, 39-56 (2012).

23. Romandini, M., Bertola, S. & Nannini, N. Nuovi dati sul Paleolitico dei Colli Berici: risultati preliminari dello studio archeozoologico e delle materie prime litiche della Grotta del Buso Doppio del Broion (Lumignano, Longare, Vicenza). *Studi di Preistoria e Protostoria* 2, 53-59 (2015).
24. Carrera, L., Pavia, M., Peresani, M. & Romandini, M. Late Pleistocene fossil birds from Buso Doppio del Broion Cave (North-Eastern Italy): implications for palaeoecology, palaeoenvironment and palaeoclimate. *Bollettino della Società Paleontologica Italiana* 57, 145-174 (2018).
25. Petrović, J. *Jame i pećine SR Srbije.* Vojnoizdavački zavod (Beograd 1976).
26. Dimitrijević, V. Upper Pleistocene mammals from cave deposits of Serbia. *Geološki anali Balkanskog poluostrva* 61.2, 179-370 (1997).
27. Đurović, P. (ed.). Speleological Atlas of Serbia. *Geographical Institute of the Serbian Academy of Sciences and Arts "Jovan Cvijić", Special Issues 52* (Belgrade 1988).
28. Kaludjerović, Z. Paleolithic in Serbia in the Light of the recent Research. *Starinar* 42, 1-8 (1993).
29. Hedges, R. E. M., Housley, R. A., Bronk, C. R. & van Klinken, G. J. Radiocarbon dates from Oxford AMS system: Archaeometry Datelist 11. *Archaeometry* 32, 211–237 (1990).
30. Dimitrijević, V. Quaternary Mammals of the Smolućka cave in Southwest Serbia. *Palaeontologia Jugoslavica*, 41-84 (1991).
31. Dimitrijević, V. Results of Investigating Vertebrate Fauna from the Paleolithic Habitat of Smolućka Cave near Novi Pazar. *Starinar* 42, 9-17 (1993).
32. Mihailović, D., Đuričić, Lj. & Kaluđerović, Z. Istraživanje paleolita na području istočne Srbije. In: *Arheologija istočne Srbije, naučni skup Arheologija istočne Srbije*, Beograd – Donji Milanovac, decembar 1995 (ed. Lazić, M.), 33-42 (Beograd 1997).

33. Rabeder, G., Hofreiter, M., Nagel, D. & Withalm, G. New Taxa of Alpine Cave Bears (Ursidae, Carnivora). *Proceedings of the 9th International Cave Bear Conference, Cahiers scientifiques du Centre de Conservation et d'Etude des Collections (Muséum d'Histoire naturelle de Lyon) / Hors Série* 2, 49-67 (2004).

34. Knapp, M., Rohland, N., Weinstock, J., Baryshnikov, G., Sher, A., Nagel, D., Rabeder, G., Pinhasi, R., Schmidt, H. A. & Hofreiter, M. First DNA sequences from Asian cave bear fossils reveal deep divergences and complex phylogeographic patterns. *Mol Ecol. 18*, 1225-1238, doi:10.1111/j.1365-294X.2009.04088.x (2009).

35. Stiller, M., Molak, M., Prost, S., Rabeder, G., Baryshnikov, G., Rosendahl, W., Münzel, S., Bocherens, H., Grandal-d'Anglade, A., Hilpertl, B., Germonpré, M., Stasyk, O., Pinhasi, R., Tintori, A., Rohland, N., Mohandesan, E., Ho, S. Y. W., Hofreiter, M. & Knapp, M. Mitochondrial DNA diversity and evolution of the Pleistocene cave bear complex. *Quaternary International 339–340*, 224-231, doi:10.1016/j.quaint.2013.09.023 (2014).
